# Trends and geospatial distribution of stillbirths in Uganda, 2014–2020

**DOI:** 10.1186/s12884-024-06434-x

**Published:** 2024-04-08

**Authors:** Petranilla Nakamya, Allan Komakech, Stella M. Migamba, Claire Biribawa, Benon Kwesiga, Lilian Bulage, Alex R. Ario, Felix Ocom

**Affiliations:** 1 Uganda National Institute of Public Health, Kampala, Uganda; 2 National Public Health Emergency Operations Centre, Ministry of Health, Kampala, Uganda

**Keywords:** Stillbirth, Deliveries, Foetus, Pregnancy, Trends, Distribution, Uganda

## Abstract

**Introduction:**

Uganda with 17.8 stillbirths per 1,000 deliveries in 2021, is among the countries with a high burden of stillbirths globally. In 2014, Uganda adopted the World Health Organization Every New-born Action Plan (ENAP), which targets < 10 stillbirths per 1,000 deliveries by 2035. Little is known about the trends of stillbirth burden since ENAP was introduced. We assessed the temporal, and spatial distribution of stillbirths, in Uganda, 2014–2020, to inform programming for safe pregnancies and deliveries.

**Methods:**

We obtained and analysed stillbirth surveillance data from the District Health Information System, 2014–2020. A stillbirth was defined as the death of a foetus > 28 weeks of pregnancy or weighing > 1000 g before or during birth and reported to a health facility. We calculated annual incidence rates of stillbirths per 1,000 deliveries at district, regional, and national levels. We used logistic regression to determine the significance of trends.

**Results:**

The overall national annual incidence of stillbirths decreased from 24/1,000 deliveries in 2014 to 17/1,000 deliveries in 2020. During the same period, reporting rates declined from 71% in 2014 to 46% in 2020. The central region continuously had the highest incidence rate for the past 5 years despite the largest decline (OR = 0.79; CI = 0.77–0.83, *P* < 0.001) while the eastern region had the smallest decline (OR = 0.59; CI = 0.57–0.61, *P* < 0.001). Districts with persistently high annual incidence rates of stillbirths (> 30/1000) included Mubende, Kalangala, Hoima, and Nebbi. There was no difference in the reporting rates of the most- vs. least-affected districts.

**Conclusion:**

Even with suboptimal reporting, the incidence of stillbirths remained above the national target. Specific areas in the country appear to have particularly high stillbirth rates. We recommend continuous capacity building in managing pregnant women with an emphasis on the most affected districts, and investigation into the reasons for low reporting.

**Supplementary Information:**

The online version contains supplementary material available at 10.1186/s12884-024-06434-x.

## Background

Stillbirth is when a baby dies after 28 weeks of pregnancy or more than 1000 g but before or during birth. Stillbirths are classified into macerated or fresh stillbirths depending on when they occur [1]. Macerated stillbirth is the intrauterine death of a foetus before the onset of labour where the foetus has shown degenerative changes while fresh stillbirth is the intrauterine death of a foetus during labour or delivery [1].

Stillbirths are a growing public health concern. The United Nations Inter-Agency Group for Child Mortality Estimation released its first-ever global stillbirth estimates in 2020, which revealed that the ratio of the number of stillbirths to the number of under-five deaths has increased from 0.77 in 2000 to 0.82 million in 2019, globally [2]. The global estimate of stillbirths is 2 million babies yearly, with three out of 4 stillbirths occurring in Sub-Saharan Africa (SSA) or Southern Asia [2]. In SSA, the stillbirth rate stands at 21.7 per 1,000 total births. Stillbirths are often underreported, so even these numbers may be underestimated [1, 3, 4].

In low- and middle-income settings, maternal conditions associated with stillbirth include hypertension, diabetes, maternal infection (e.g. syphilis, malaria, HIV), maternal undernutrition, obesity, and smoking [5]. Other factors which significantly contribute to stillbirths include: foetal asphyxia, trauma, prolonged labour, congenital infections, and foetal distress [6, 7].

With quality health care throughout pregnancy and childbirth, most stillbirths are preventable. In Uganda, the rate of stillbirths in 2015 was 21/1,000 live births. In 2021, a study in a hospital in the Northern part of Uganda showed a stillbirth rate of 20 deaths per 1,000 deliveries [8]. The government of Uganda has ensured that there are health facilities within every 5 km radius to help mothers easily access healthcare.

 [9], it has also provided free antenatal care services where mama-kits are distributed to help mothers and their unborn babies during delivery [10]. Public health facilities also provide folic acid and iron supplementation, prevention of malaria through providing intermittent preventive treatment and distribution of treated mosquito nets, and improved detection and management of syphilis to pregnant women to improve pregnancy outcomes.

In 2014, the World Health Organisation developed an action plan to prevent stillbirths. This plan, called the Every Newborn Action Plan (ENAP), was adopted by Uganda and targets a reduction in stillbirth rates to < 12 per 1,000 total births by 2025 and < 10 stillbirths per 1000 total births by 2035 [11]. The plan involves supporting government leadership and providing guidance on how to strengthen newborn health components in existing health sector plans and strategies, especially those that relate to reproductive, maternal, and child health. However, little is known about stillbirth rates in Uganda since ENAP was introduced. We assessed the distribution, temporal, and spatial trends of stillbirths in Uganda, 2014–2020, to inform programming for safe pregnancies and deliveries in the country.

## Methods

### Study setting

This was a nationwide study in Uganda, East Africa. Uganda had approximately 47 million people in 2021 and her fertility rate was 4.7 births per woman in 2020 [12]. The health service delivery is organized in levels from the lowest; Health Centres two (HC11), three (HCIII), four (HCIV), general hospital, regional referral hospital, and national referral hospital which is the highest. All health centres ideally should provide maternal and childcare services, however, the range of services advances with the level of the health centre. Health centre two(s) are limited to immunization and antenatal care, health centre three(s) include the above plus admissions for maternity. Health centre four(s) have admission wards and caesarean section services, whereas hospitals additionally have neonatal units. Health facilities in Uganda are either public or private ownership. A number of private clinics are not registered in the DHIS2 system, and hence, do not report on the system. Additionally, some registered private clinics mostly those on the level of HCII still have very poor reporting rates or do not report at all. These were excluded from the data analysed. Exceptionally, we have a significant number of traditional birth attendants. These are informal and hence do not report on the District Health information system.

### Study design and data source

We conducted a descriptive analysis of still-births surveillance data reported through the electronic District Health Information System (DHIS2), a computer-based national health database. The surveillance case definition for stillbirth is the death of a foetus weighing > 1000 g or > 28 weeks of pregnancy, either before or during birth. Data on stillbirths is routinely generated at registered health facilities (all public health facilities plus most private health facilities), aggregated at the district level, and then forwarded to the national database. This data is then analysed and interpreted by authorized Ministry of Health officials and other stakeholders to guide public health action towards stillbirths.

### Study variables and data analysis

We abstracted data using pivot tables in DHIS2 on still-births as well as total deliveries in the health facilities from January 2014 to December 2020. We obtained data on fresh stillbirths, macerated stillbirths, and total deliveries. The data from DHIS2 was downloaded, merged, and summarized in Microsoft Excel sheets.

Data on fresh and macerated stillbirths were summed to obtain the total stillbirths. We calculated incidence rates for stillbirths by country, region, district, and year. We calculated the annual incidence rates by dividing the total number of still-births by the total deliveries in health facilities, multiplied by 1,000 in Uganda between 2014 and 2020. We obtained mean annual incidence rate by summing the annual incidence rate divided by seven. The incidence rates were presented on a trend line graph. The total stillbirths were disintegrated into Fresh stillbirths (FSB) and Macerated stillbirths (MSB). We calculated the proportion of FSB out of the total stillbirths. The incidence rate of FSB was equal to the number of FSB divided by total deliveries, multiplied by 1,000 while the incidence rate of MSB was equal to the number of MSB divided by total deliveries, multiplied by 1,000. We also abstracted data on reporting rates to ascertain the completeness of the data. Each health facility has a mandate of submitting monthly reports to be entered into DHIS2. These are automatically aggregated by the system to give annual reporting rates.

We then imported data into Epi info version 7 to do a logistics regression analysis to determine the significance of the trends. The aggregated data on still births and total deliveries for each year and region was used to obtain the number of live births. The stillbirths were coded 1 while livebirths were coded 0. This data was entered into Epi-info, and analyzed using the logistic regression gadget. We created dummy variables for time, to obtain significance of the change.

We drew choropleth maps using Quantum Geographic Information System (QGIS) to show the distribution of incidence rates of still-births at the national level in the different districts.

## Results

### Trend of the annual incidence rate of stillbirths, Uganda, 2014–2020

**Fig. 1:**
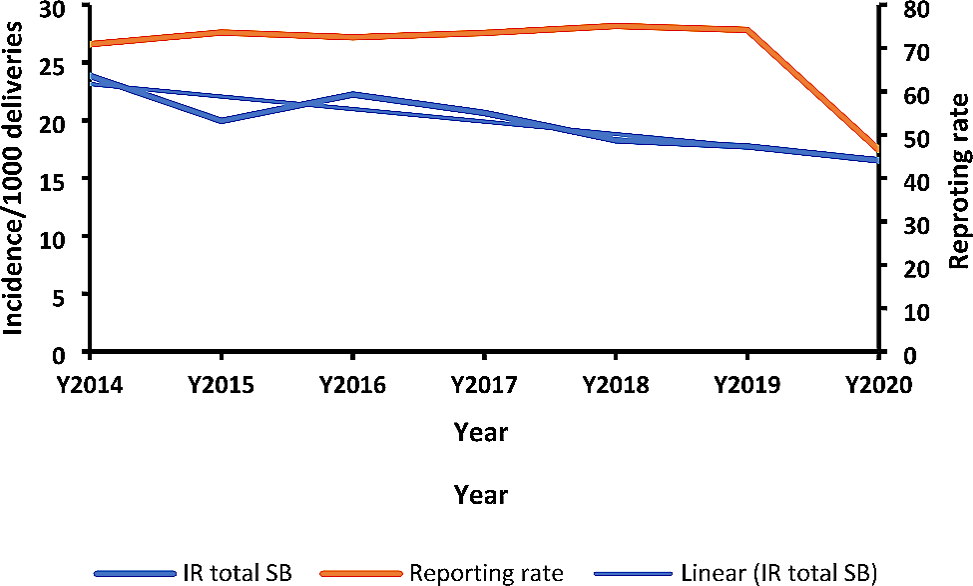
Trend of the annual incidence of stillbirths per 1,000 total deliveries, Uganda, 2014–2020

The annual incidence rate of stillbirths per 1,000 deliveries as recorded by health facilities in Uganda from 2014 to 2020 showed a decline. The mean annual incidence rate over the years was 20 stillbirths per 1,000 deliveries. The highest annual incidence over the seven years was recorded in 2014, while the lowest was in 2020. The incidence reduced from 24 stillbirths in 2014 to 20 stillbirths in 2015 and then increased to 22 stillbirths in 2016. The following years from 2017 to 2020 had an annual decline in stillbirths from 21 to 17 per 1000 deliveries (Fig. 1). The reporting rates ranged from 71 to 74% for the period 2014 to 2019; then had a steep decline to 46% in 2020.

The incidence of stillbirths decreased by 31% from 2014 to 2020 and the decreasing trend was statistically significant. (OR = 0.69; CI = 0.67–0.70, *P* < 0.001).

**Fig. 2:**
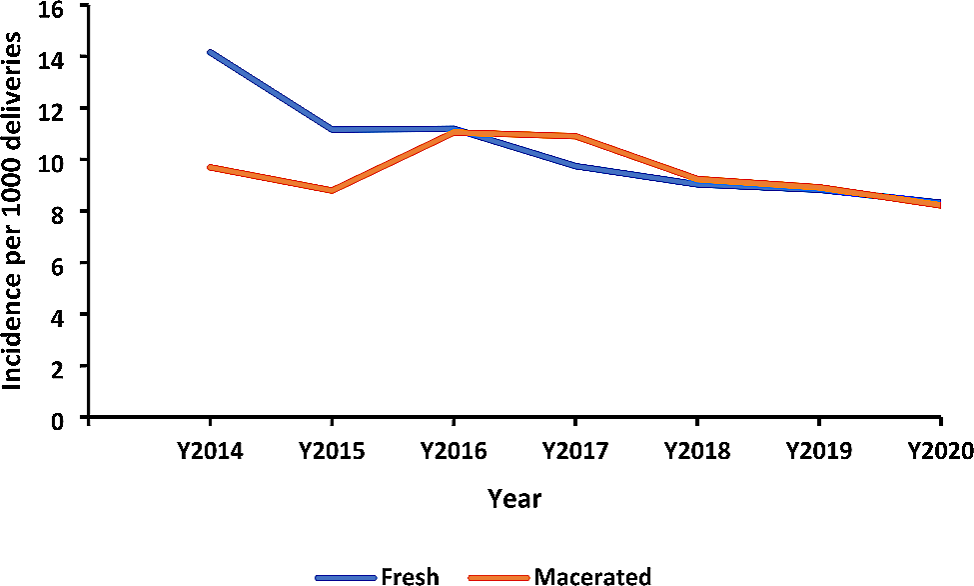
Annual incidence of fresh and macerated stillbirths per 1000 deliveries, Uganda, 2014–2020

Over the study period, the incidence rate of fresh stillbirths was slightly more than that of macerated stillbirths. In 2014 the incidence of FSB appeared much higher than that of MSB and over the following years, the difference narrowed down (Fig. 2).

### Temporal trends of still-births incidence, regional level, Uganda, 2014–2020

**Table 1:** Significance of the trends of incidence of stillbirths per 1000 deliveries, by region, Uganda, 2014–2020

Region	Odds Ratio	95% CI	P-Value
Central Region 2014/2020	0.79	0.77–0.83	< 0.001
Eastern Region 2014/2020	0.59	0.57–0.61	< 0.001
Northern Region 2014/2020	0.78	0.75–0.81	< 0.001
Western region 2014/2020	0.69	0.66–0.72	< 0.001

We observed a statistically significant decrease in the incidence rates of stillbirths per 1,000 deliveries in all the regions of Uganda (Table 1).

The eastern region had had the highest decline of still births of 41% from 2014 to 2020, while the central region had the lowest decline of 21% from 2014 to 2020. The decline rate for northern region was 22% while that for the wester region was 31%, from 2014 to 2020.

**Fig. 3:**
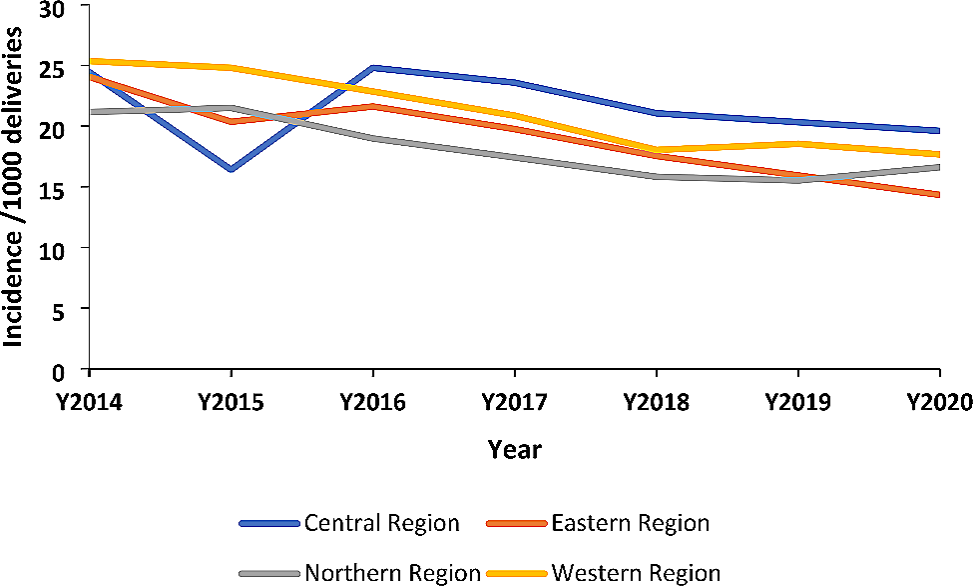
Trend of annual incidence of stillbirths per 1,000 total deliveries by region, Uganda, 2014–2020

Central and western regions had higher incidence rates of stillbirths compared to the northern and eastern regions.

The central and western regions registered the highest mean annual incidence rate of 22 still-births per 1,000 deliveries over the seven years while the Northern and Eastern regions registered the lowest mean incidence rate of 18 per 1,000 deliveries over the seven years. The central region had a steep decline in incidence in 2015 and then a rise in 2016, this was followed by a shallow decline in the subsequent years until 2020 (Fig. 3).

### Distribution of annual still-births incidence, district level, Uganda, 2014–2020

**Fig. 4:**
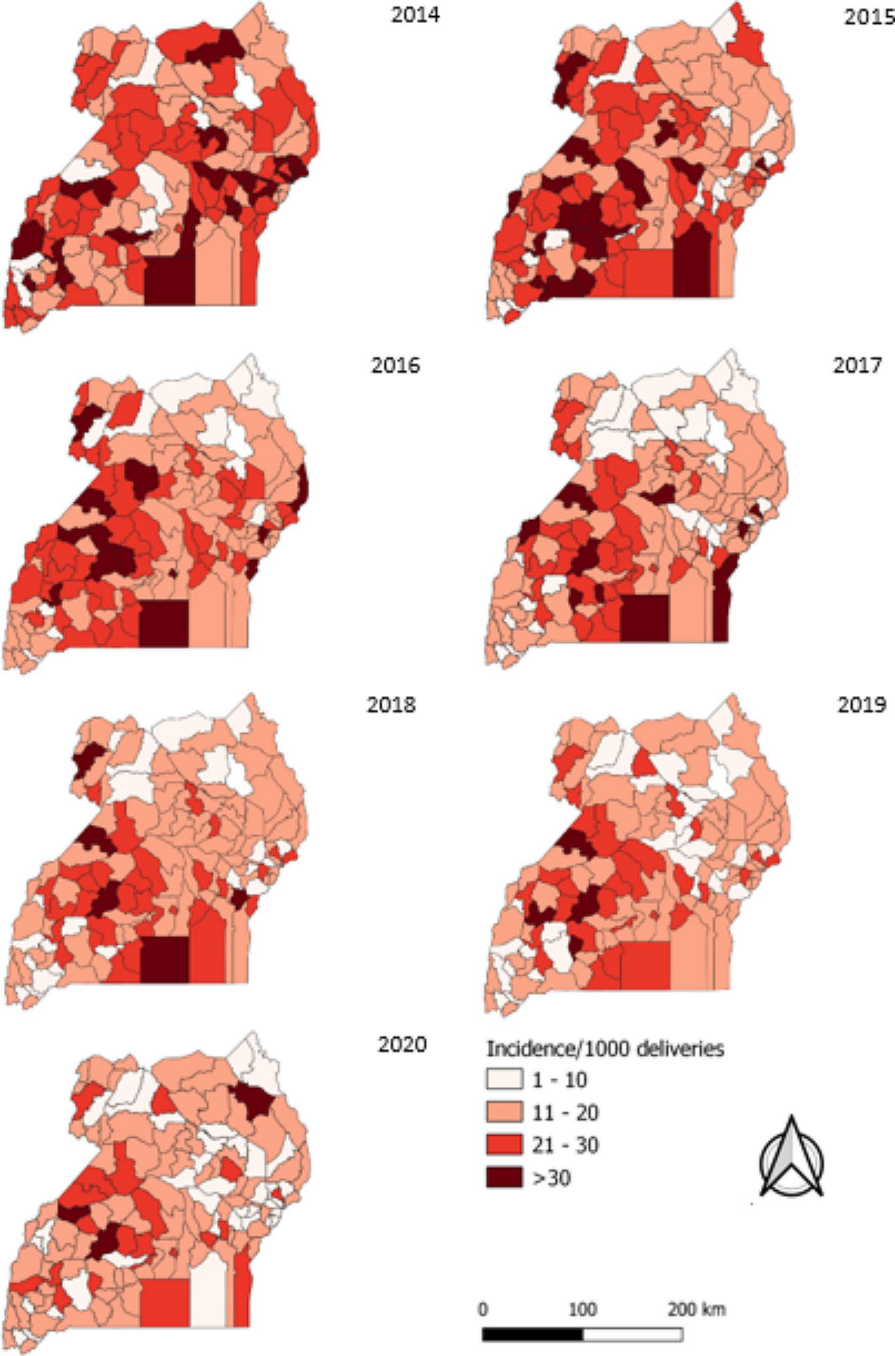
Distribution of annual stillbirth incidence per 1000 deliveries by districts, Uganda, 2014–2020

Most of the districts in Uganda had an incidence above the target − 10 stillbirths per 1,000 deliveries. Generally, there was a minimal decrease in the distribution of stillbirths from 2014 to 2020. About 20 districts registered over 30 stillbirths per 1,000 deliveries in 2014 and 2015, this number reduced in 2016. In 2017, about 10 districts had over 30 stillbirths per 1,000 deliveries. There were less than 10 districts with over 30 stillbirths in 2019 and 2020. The districts with a persistently high incidence of > 30 stillbirths per 1,000 deliveries included: Mubende, Kalangala, Hoima, and Nebbi (Fig. 4).

## Discussion

We assessed the distribution, temporal, and spatial trends of stillbirths in Uganda, 2014–2020. The highest annual incidence over the seven years was recorded in 2014, while the lowest was in 2020. Over the study period, the mean stillbirth rate was 20 stillbirths per 1,000 deliveries. Reporting on stillbirths was less than the national target of 80% throughout the study period with 2020 being the worst year. There was a significant decrease in the incidence rate of stillbirths from 2014 to 2020 in Uganda at the national, and regional levels. This is similar to what has been recorded at the global level where there has been a reduction in the stillbirth rate from 2000 to 2019, estimated at 2.3% even though the stillbirths were increasingly concentrated in Sub-Saharan Africa [13, 14].

The highest recorded incidence in the seven years happened in 2014. This is the year ENAP was initiated in Uganda. Through the ENAP initiative, health workers across the country were trained in managing deliveries and danger signs of pregnant women. Some of the factors attributed to these deaths included: the delay of the mother to seek help from a professional health worker, and the absence of critical human resources and equipment in health facilities. The causes of death included respiratory distress syndrome, birth asphyxia, prematurity, and syphilis [15]. By 2019, countries such as South Sudan, the Democratic Republic of Congo, Chad, Guinea, and Somalia had more than 25 stillbirths per 1000 deliveries while Uganda had 18 stillbirths per 1,000 deliveries [13].

The incidence of fresh stillbirths was more than that of macerated stillbirths. Similarly, a study in a peri-urban district in Ghana revealed higher fresh stillbirths which were associated with mothers with a parity of 1.6 ± 1.9 compared to mothers with macerated stillbirths with a parity of 2.54 ± 2.7 [7]. Fresh stillbirths are associated with gaps in care during labor and at delivery while macerated stillbirths are often associated with insults in utero during the antenatal period [16, 17]. Key effective interventions to reduce stillbirths include basic and comprehensive emergency obstetric care [18].

The rates of reporting on stillbirths were below the target (80%) for each of the years and this affected the representation of the actual burden of stillbirths. The year 2020 had the lowest recorded stillbirths with the lowest reporting rate. The low reporting rate shows that in 2020 the stillbirths could have been an under-estimate due to the challenges in maternal and child health services delivery caused by the response to the COVID 19 pandemic. This could be attributed to the restrictions on the movement of people and vehicles which caused a decrease in services delivered to women as well as delays in seeking care [19–21].

Many stillbirths are potentially preventable and the most common cause is placental insufficiency, followed by maternal medical disorders, hypertensive conditions, and spontaneous preterm birth [22, 23].

### Study limitations

We utilized secondary data, which is limited in terms of variables to comprehensively assess the stillbirth challenge in Uganda. The overall rate of reporting in the system was less than 80% for all years; this limited us from getting the full representation of the incidence of stillbirths in Uganda. The estimate we are reporting may be under-estimated.

Additionally, some private health facilities especially those on the level of HCII and traditional birth attendants are do not report on the DHIS2. This implies there are missed cases from these sites.

## Conclusion

The stillbirth incidence rate in Uganda remains above the national target for ENAP goals. Specific districts appear to have particularly high stillbirth rates over the study period. We recommend continuous capacity building in managing pregnant women with an emphasis on the most affected districts, and investigation into the reasons for low reporting.

### Electronic supplementary material

Below is the link to the electronic supplementary material.

Supplementary Material 1

## Data Availability

The dataset used for this study are a property of the Uganda MOH and are not publicly available. However, with a reasonable request and permission from the Uganda MOH, data can be accessed through the corresponding authors or from the DHIS2.

## Declarations

All protocols for this project were reviewed in line with the CDC policy by the US CDC human subjects review board in accordance with the Declaration of Helsinki. All methods were carried out in accordance with relevant guidelines and regulations.

## Abbreviations

COVID: Corona Virus Disease
DHIS2: District Health Information System
ENAP: Every Newborn Action Plan
FSB: Fresh Stillbirth
HC: Health Centre
MOH: Ministry of Health
MSB: Macerated Stillbirth
QGIS: Quantum Geographic Information System
SSA: Sub-Saharan Africa
WHO: World Health Organisation
